# Molecular characterization and prevalence of *Halarachne halichoeri* in threatened southern sea otters (*Enhydra lutris nereis*)

**DOI:** 10.1016/j.ijppaw.2018.09.009

**Published:** 2018-09-29

**Authors:** Risa Pesapane, Erin Dodd, Nadia Javeed, Melissa Miller, Janet Foley

**Affiliations:** a School of Veterinary Medicine, Department of Medicine and Epidemiology, University of California Davis, 1320D Tupper Hall, Davis, CA 95616, United States; b Marine Wildlife Veterinary Care and Research Center, California Department of Fish and Wildlife, 151 McAllister Way, Santa Cruz, CA, 95060, United States

**Keywords:** Halarachnidae, Sea otter, Acarology, Marine parasites, *Enhydra lutris*, *Halarachne halichoeri*

## Abstract

Parasitism, particularly in concert with other sublethal stressors, may play an important, yet underappreciated role in morbidity and mortality of threatened species. During necropsy of southern sea otters (*Enhydra lutra nereis*) from California submitted to the Marine Wildlife Veterinary Care and Research Center's Sea Otter Necropsy Program between 1999 and 2017, pathologists occasionally observed nasopulmonary mites infesting the respiratory tracts. Infestation was sometimes accompanied by lesions reflective of mite-associated host tissue damage and respiratory illness. Our objectives were to estimate prevalence of nasopulmonary mites, determine the taxonomic identity of the observed mites, and create a DNA reference for these organisms in southern sea otters as an aid in population management. Using unique morphological characteristics discerned via light and scanning electron microscopy (SEM), we identified the mites as *Halarachne halichoeri,* a species typically associated with harbor seals (*Phoca vitiluna*). The 18S, 16S, 28S and ITS1-2 genetic regions were sequenced and submitted to GenBank. We observed *H. halichoeri* mites in 25.6% (95% CI 19.9–33.4%). of southern sea otters from a subset of necropsies performed between 2012 and 2017. This is the first documentation of *H. halichoeri* in southern sea otters and is suggestive of parasite exchange between sea otters and harbor seals.

## Highlights

• Southern sea otters (*Enhydra lutris nereis*) harbor the mite *Halarachne halichoeri*.
• 25.6% of southern sea otters necropsied from 2012 to 2017 harbored respiratory mites.
• Larval halarachnid mites may live up to 3 weeks outside of any host.
• Sequence for the 28S, ITS1-2, 18S, and 16S of *Halarachne halichoeri* were generated.

## Graphical abstract

**Figure:**
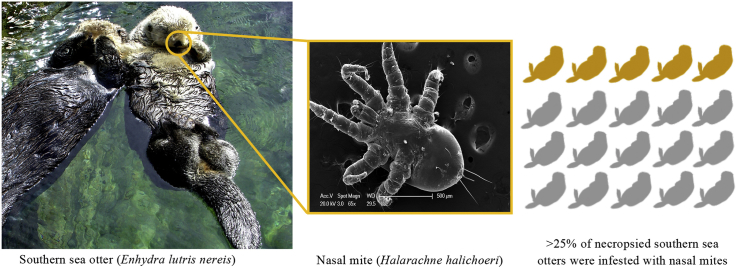

## 1 Introduction

The southern sea otter (*Enhydra lutris nereis*) is a federally protected species (USFWS, 1977) that is found only in California, USA. Deceased sea otters found along the California coast are examined at the California Department of Fish and Wildlife Marine Wildlife Veterinary Care and Research Center (MWVCRC) in Santa Cruz, CA in order to determine causes of death. Of particular importance is assessing potential sub-lethal threats that could contribute to morbidity and mortality. One potential threat is invasion of the respiratory tract by nasopulmonary mites, because these parasites can cause tissue damage and respiratory illness (Kenyon et al., 1965; Dunlap et al., 1976; Kim et al., 1980; Baker, 1987; Alonso-Farré et al., 2012).

Mites known to parasitize the respiratory tracts of marine mammals encompass two genera of the family Halarachnidae (Acari: Mesostigmata) (Mullen and O'Connor, 2002): *Orthohalarachne* mites are commonly associated with fur seals (Otariidae) and walrus (Odobenidae), whereas *Halarachne* mites are typically found in earless seals (Phocidae) but have been reported in non-pinnipeds, including northern sea otters (*Enhydra lutris kenyoni*) (Domrow, 1962; Kenyon et al., 1965). Mites of both genera can cause respiratory illness, including sinusitis, sneezing, coughing, facial pruritus, head shaking, pneumonia, edema, and lung congestion (Dunlap et al., 1976; Baker, 1987; Alonso-Farré et al., 2012). In a sample of necropsied southern sea otters, nasopulmonary mite burden was reportedly mild (<10 mites) to heavy (>50 mites), and sea otter infestation was significantly associated with upper respiratory inflammation, aged adult age class, captive care within 10 days of death, and stranding near a large brackish slough that was heavily utilized by sympatric sea otters and harbor seals (Shockling-Dent et al., submitted to IJPPAW). This same manuscript also provides insight into associations among common respiratory findings and nasopulmonary acariasis in necropsied southern sea otters.

Neither the prevalence nor taxonomy of nasopulmonary mites in southern sea otters has been investigated. Therefore the extent and potential origin of mite infestations in this population remain understudied yet imperative components for understanding the impact of nasopulmonary mites in southern sea otters. Here we present prevalence data on respiratory mite infestations in a sample of southern sea otters, clarify the species of respiratory mites infesting southern sea otters, and provide the first DNA sequence data for mites from the family Halarachnidae.

## 2 Material and methods

Respiratory mites were obtained from southern sea otters during detailed necropsies at MWVCRC (Kreuder et al., 2003). Using flat-bladed instruments, mites were scooped into cryovials during gross necropsy when observed on the planum nasale, rostral nose, turbinates, nasopharynx, trachea and/or bronchi. Small, highly motile “crab-like” mites from the planum nasale, rostral nose or turbinates were often collected and pooled as a single aliquot, and more sessile mites with elongated, “cigar-shaped” bodies from the nasopharynx, trachea and/or bronchi were collected and pooled as a separate aliquot. Sampled mites were immediately fixed in 70% ethanol or stored at −20 °C prior to placement in 70% ethanol, or stored at −20 °C prior to placement in 70% ethanol, and a single sample of live mites were stored in 0.9% saline at 4 °C.

Whole mites preserved in 70% ethanol were examined at 10X-100X on a light microscope to assess morphology and identify genus-specific anatomic features. Scanning electron microscopy (SEM) was also used to facilitate species identification based on published morphological criteria (Furman and Dailey, 1980). Sample dehydration for SEM was accomplished via immersion of mites in increasing concentrations of ethanol (in triplicate) through 100%, followed by critical point drying in a Tousimis 931.GL Autosamdri critical point dryer (Tousimis Research Corp., Rockville, Maryland). Dehydrated mites were mounted on stubs and sputter-coated with gold using a PELCO SC-7 coater (Ted Pella, Redding, California). Mites were examined and photographed on an FEI XL30 TMP scanning electron microscope (Eindhoven, The Netherlands).

At present, there is insufficient published molecular data available to facilitate detailed molecular characterization of *Halarachnidae* sp. mites. To begin to develop a preliminary DNA reference for the organism obtained from southern sea otters, the 18S, 16S, 28S, and ITS1-2 genetic regions were sequenced as follows: DNA was extracted from individual mites by heating at 56 °C for 15 min to evaporate ethanol. Mites were pierced with a sterile needle to facilitate DNA extraction, followed by DNA isolation using the QIAmp DNA Micro Kit (Qiagen, Hilden, Germany) according to manufacturer instructions. PCR primers were used in a 25-μL volume PCR reaction containing GoTaq Green Master Mix (Promega, Madison, WI), with cycling conditions as previously described (Black and Piesman, 1994; Morelli and Spicer, 2007; Dowling and Oconnor, 2010; Foley et al., 2013). PCR products were visualized on a 1% agarose gel and purified using ExoSAP-IT (ThermoFisher, West Sacramento, CA) before sequencing on an ABI 3730 sequencer (UCDNA Sequencing Facility, Davis, CA) using the forward primer. Sequenced amplicons were evaluated by BLAST search of GenBank (NCBI; http://blast.ncbi.nlm.nih.gov/Blast.cgi).

Nucleotide sequence data reported in this paper are available in the GenBank™ database under the accession numbers: **MH426929, MH426930, MH426846, MH426847, MH426849, MH426845, MH426848**. The Parasitology Unit of the National Veterinary Services Laboratories (United States Department of Agriculture, Animal and Plant Health Inspection Service, Veterinary Services, Science, Technology, and Analysis Services) in Ames, IA, confirmed identity of the mites morphologically, and voucher specimens are archived there as “General cases” under accession numbers **17-023810, 17-035319 to 17-035322, 17-035324, and 17-035325**.

To assess the prevalence of mite infestations, the MWVCRC electronic database was queried using the following criteria to create a sample set with reduced possibility of false negatives: sea otters that stranded from 2012 through 2017; carcasses were fresh never frozen; and where detailed necropsy included examination of the nares, nasopharynx and oropharynx by staff who were trained to recognize mites. Using the same described criteria to define negative controls, all visually-confirmed, mite-positive carcasses examined during this same timeframe were included as positive controls. Prevalence and 95% confidence intervals were calculated using the function *prop.test* in base R version 3.4.3 (R Core Team, 2017).

## 3 Results

### 3.1 Demographic parameters

Among 156 southern sea otters necropsied between 2012 and 2017 that matched our query criteria, 40 harbored respiratory mites for an estimated prevalence of 25.6% with 95% CI 19.9–33.4%.

### 3.2 Morphological identification

**Fig. 1:**
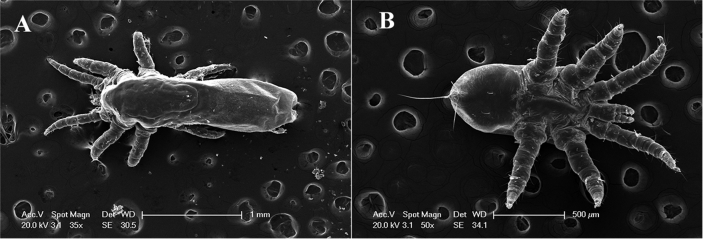
(A) Scanning electron microscopy of adult *Halarachne halichoeri* showing opisthosoma (abdomen) with slight constriction only at the anterior end and dorsal shield broader posteriorly than anteriorly with linguiform caudal tip. (B) Scanning electron microscopy of larvae *Halarachne halichoeri* with postanal setae (bristles) longer than adanal setae.

A total of 213 mites collected from 23 sea otters necropsied between 2007 and 2017 were used for taxonomic identification. Microscopic examination of mites documented “crab-like” hexapod larvae, and “cigar-shaped” octopod adults belonging to the genus *Halarachne*, with all specimens exhibiting anatomical features matching a prior description of *H. halichoeri* (Furman and Dailey, 1980). Adults (Fig. 1A) demonstrate: subcylindrical or saccate opisthosoma (abdomen) with slight constriction only at the anterior end as opposed to clavate with abrupt constriction of the posterior end distinguishing them from *H. lysanae;* dorsal shield broader posteriorly than anteriorly with linguiform caudal tip as opposed to being broader anteriorly than posteriorly with narrow caudal tip as in *H. miroungae*. Larvae (Fig. 1B) demonstrate: postanal setae (bristles) longer than adanal setae.

### 3.3 Molecular characterization

PCR of individual mites yielded amplicons ranging from 245 to 587bp of the 16S, 18S, ITS1-2, and 28S regions. Primers, amplicons, and BLAST results are summarized in Table 1. Across all four genetic regions, our *H. halichoeri* mites showed 78-98% homology with other mesostigmatid mite families from the superfamily Dermanyssoidea. Importantly, data for *H. halicoeri* cover five different genetic regions, and show that, while homology with the *Ornithonyssus, Ichoronyssus, and Dinogamasus* genera is very high (98%) for 18S, other genera have greater homology for other genetic regions, but none greater than 91%.

**Table 1:** Sequencing results of PCR amplicons from four genetic regions of *Halarachne halichoeri* from southern sea otters, *Enhydra lutris nereis,* from California.

Genes	Primers	Sequences (5‘-3‘)	Source	Amplicon size (bp)	BLAST Results (percent homology)
18S	F: 31F1 R: 344R2	CGCGAATGGCTCATTAAATC GCCTTCCTTGGATGTGGTAG	Foley et al. (2013)	289	*Dinogamasus 98%,* *Ichoronyssus 98%,* *Ornithonyssus 98%*
16S I	F: 16S + 1 R: 16S-2	CTGCTCAATGATTTTTTAAATTGCTGTGG TTACGCTGTTATCCCTAGAG	Black and Piesman (1994)	266–267	*Stylochyrus 78%*
16S II	F: 16S + 2 R: 16S-1	TTGGGCAAGAAGACCCTATGAA CCGGTCTGAACTCAGATCAAGT	Black and Piesman (1994)	245–246	*Ornithonyssus 85%,* *Dermanyssus 84%*
28S	F: 43F R: 929R	GCTGCGAGTGAACTGGAATCAAGCCT AGGTCACCATCTTTCGGGTC	Dowling and Oconnor (2010)	440–587	*Gaeolaelaps* 92%, *Cosmolaelaps* 91%
ITS1-2	F: ITS-1F R: ITS-1R I: ITS-1int	AGAGGAAGTAAAAGTCGTAACAAG ATATGCTTAAATTCAGGGGG GGTCTTCACATYTGATTTCAG	Morelli and Spicer (2007)	469–475	*Coleolaelaps* 89%, *Tropilaelaps* 89%

### 3.4 Incidental mite survival

Two larval mites from a single fresh sea otter carcass were harvested alive and stored in saline solution. One mite was used for microscopy, and the other was held in saline solution and checked daily for viability. The mite held in saline stored at 4 °C survived for 22 days with no nutritional supplementation.

## 4 Discussion

Although necropsy records at the MWVCRC document southern sea otters infested with nasopulmonary mites at least as far back as 1999 (unpub. data Miller), the identity, prevalence, pathology, and ecology of these mites remained unstudied over the past two decades. Here we provide evidence for nasopulmonary mite infestation in a quarter of our sample of necropsied southern sea otters, establishing a new host record for *H. halichoeri.* We also provide the first DNA sequences for any member of the family Halarachnidae. In view of the sparse coverage in the GenBank database for mites in the mesostigmatan superfamily Dermanyssoidea, beyond those associated with apiculture, our sequences provide baseline data to facilitate future studies evaluating genetic diversity among these mites, develop molecular diagnostic tools, and could help document interspecific mite infestations.

*Halarachne halichoeri* was originally described as a respiratory parasite of gray seals (*Halichoerus grypus*) (Allman, 1847), but the literature on this mite has been fraught with misidentification, controversy, and repeated taxonomic turmoil. Along the Pacific Coast of North America, respiratory mites collected from harbor seals (*Phoca vitiluna*) from Pacific Grove, CA, in 1923 were originally identified as *H. otariae* (Ferris, 1925), but were later reclassified as *H. halichoeri* (Ferris, 1942). Newell (1947) cast doubt on this identification due to lack of other evidence that *H. halichoeri* existed off the Pacific Coast, and suggested that any mites of this morphology in the Pacific Ocean realm should be classified as *H. miroungae.* As a result, respiratory mites obtained from both captive and wild sea otters were reported as *H. miroungae (sensu lato)* into the 1960s (Newell, 1947; Kenyon et al., 1965). Although Furman and Dailey (1980) later concluded that sea otter respiratory mites were rightly *H. halichoeri*, it seems that this determination was based on knowledge of technical errors in the pre-existing species description, and not based on re-examination of actual specimens. Our study conclusively demonstrates sea otters as hosts for *H. halichoeri*.

Hosts of *Halarachne* spp. mites were thought to be strictly members of the pinniped family Phocidae, until a captive gentoo penguin (*Pygoscelis papua*) and a captive sea otter housed proximate to phocids were found to be infested (Domrow, 1962; Kenyon et al., 1965). Kenyon et al. (1965) necropsied 200 wild northern sea otters from Amchitka Island, AK, and found *Halarachne* respiratory mites in 3%, confirming that these infestations were not merely a product of proximity to infested seals in captivity. Although northern and southern sea otters share their habitat with a diverse range of respiratory mite-infested pinnipeds, including northern elephant seals (*Mirounga angustirostris*) infested with *H. miroungae*, and California sea lions (*Zalophus caliofornianus*) infested with *Orthohalarachne* spp., sea otters only appear to become infested with *Halarachne* spp. mites associated with seals of the genus *Phoca.* This could reflect host preferences among mite parasites. However, marine halarachnid infestations have been documented in pinnipeds, otters, marine birds, and even humans (Domrow, 1962; Kenyon et al., 1965; Webb et al., 1985), suggesting that these mites may be opportunistic rather than preferential regarding hosts that they infest. Groups of harbor seals and southern sea otters are often observed resting on the same beaches and rocky outcrops in California, providing possibilities for interspecific parasite transmission. Interestingly, the coastal area with the highest concentration of sea otters and harbor seals resting in close proximity to each other (Elkhorn Slough) was a high-risk site for nasopulmonary acariasis for 209 necropsied southern sea otters in a recent study (Shockling-Dent et al., submitted to IJPPAW). In that study, necropsied otters stranding within 1 km of the slough were 4.9 times more likely to have nasopulmonary acariasis. However, additional factors may have contributed to the observed risk, including a relatively high sea otter density, and a comparatively high proportion of released otters with a history of captive care (Shockling-Dent et al., submitted to IJPPAW). Spatial, biotic, and environmental relationships between Pacific seals and sea otters, and any consequent potential for parasite exchange between them, may be important and warrant further investigation.

The single human case of halarachnid mite infestation involved a tourist at a marine park who was treated for ophthalmic acariasis after standing in close proximity to a walrus exhibit where he was presumably exposed as the walrus expelled respiratory mites while snorting and spitting (Webb et al., 1985). One adult specimen of *O. attenuata* was recovered from the patient's eye after he sought medical attention for intense ocular pain and irritation associated with corneal abrasion. Although halarachnid-associated acariasis is likely extremely rare in humans, this case does demonstrate that these mites are capable of causing discomfort and damage. Those who work closely with respiratory mite-infested marine mammals should take precautions to minimize risk of transmission, particularly in zoological settings or during rehabilitation and oil spill response activities.

Halarachnid mites are thought to be transmitted directly (as motile larvae) between individual host animals in proximity (Fay and Furman, 1982; Kurochkin and Sobolewsky, 1971). However, our finding that a single *H. halichoeri* larva held in saline solution survived for three weeks demonstrates the hardiness of the larval stage outside of the host. Although this is just one mite and the storage conditions do not mimic that of California marine habitats, this incidental finding is consistent with what has been reported about the survival of *Orthohalarachne attenuata* larvae which survived for 18-27 days in saline at temperatures ranging from 21 to 27 °C after first being held for several days at 4 °C (Furman and Smith, 1973). The possibility that environmental contamination by larval mites may also facilitate parasite spread, particularly among marine mammals in captivity, should therefore not be discounted without conducting further empirical studies.

Our documentation of 25.6% *H. halichoeri* infestation in necropsied southern sea otters may underestimate the true prevalence, due to various biological and technical issues. These mites are very small and visual detection can be challenging, especially if the carcass has decomposed or has been frozen, when the mite load is low, when sand, digesta, pus, mucus or seawater is present in the respiratory tract, or when the respiratory tract is only partially examined. Larval mites can easily be missed in autolyzed carcasses because they are able to leave the carcass in search of potential new hosts. Similarly, because larvae are most common inside the nares and on the nasal turbinates, they are easily missed during routine necropsy, especially for frozen-thawed carcasses containing non-viable, non-motile mites (unpub. data Miller). The current dataset is limited to examination of necropsied otters; assessments of nasopulmonary mite prevalence in ostensibly healthy, live-captured sea otters might provide a more precise estimate of infestation in the free-ranging population. However, since endoscopic evaluation may miss mites present only in the deeper respiratory tract or in the sinuses, parallel studies of live-captured and necropsied otters is recommended.

Despite numerous reports of parasite infestations of wildlife, often an understanding of the ecology, geographic distribution, pathology, and details of parasite morphological or molecular identification is deficient. As part of the current study, we have begun to address these gaps for marine halarachnid respiratory mites. Scientific studies have highlighted the importance of mite infestations in enhancing host morbidity, as important underlying agents of injury, as potential vectors for pathogen transmission (Baker, 1946; Kim et al., 2005; Hubert et al., 2017), and for facilitating pathogen invasion (Swe et al., 2014). Collectively these findings, a complementary study on nasopulmonary mite pathology, and risk factors for sea otter infestation (Shockling-Dent et al., submitted to IJPPAW), and our conclusions from the current study, demonstrate that the potential health impacts associated with mite infestations have been under-recognized, including for sea otters. Marine respiratory mites can contribute to mucosal irritation, destruction of turbinates, respiratory illness, and in severe cases, sea otter death (Kenyon et al., 1965; Shockling-Dent et al., submitted IJPAW). Heavy burdens of respiratory mites were recently implicated in facilitating a lethal β-hemolytic *Streptococci*-associated outbreak in fur seal pups (Seguel et al., 2018). β-hemolytic *Streptococci* are important southern sea otter pathogens (Imai et al., 2009; Bartlett et al., 2016), and it is possible that halarachnid mites may spread other pathogens in sea otters. Future research should focus on further clarifying halarachnid mite ecology, improving diagnostic methods, validating treatment options for sea otters under human care, and assessing the potential for these mites to spread pathogens between phocids and sea otters, or among sympatric sea otters.

## Declarations of interest

None.

## Appendix A Supplementary data

Data Profile Data Profile

Data Profile Data Profile
